# Biliverdinuria Caused by Exonic *BLVRA* Deletions in Two Dogs with Green Urine

**DOI:** 10.3390/genes15121561

**Published:** 2024-11-30

**Authors:** Eva Furrow, Jade A. Peralta, A Russell Moore, Katie M. Minor, Candace Guerrero, Charlotte R. Hemmila, Victoria DiCiccio, Jonah N. Cullen, Steven G. Friedenberg, Urs Giger

**Affiliations:** 1 College of Veterinary Medicine, University of Minnesota, St. Paul, MN 55108, USA; 2 College of Veterinary Medicine & Biomedical Sciences, Colorado State University, Fort Collins, CO 80523, USA; 3 Center for Metabolomics and Proteomics, University of Minnesota, St. Paul, MN 55108, USA; 4 VCA West Los Angeles Animal Hospital, Los Angeles, CA 90025, USA; 5 Clinic for Small Animal Internal Medicine, Vetsuisse Faculty University of Zürich, 8057 Zürich, Switzerland; giger@vet.upenn.edu

**Keywords:** *Canis lupus familiaris*, animal model, inborn error of metabolism, whole-genome sequencing, heme catabolism

## Abstract

**Background/Objectives:** In heme degradation, biliverdin reductase catalyzes the conversion of biliverdin to bilirubin. Defects in the biliverdin reductase A gene (*BLVRA*) causing biliverdinuria are extraordinarily rare in humans, and this inborn error of metabolism has not been reported in other mammals. The objective of this study was to diagnose biliverdinuria and identify the causal *BLVRA* variants in two adult mixed-breed dogs with life-long green urine. One of the dogs also had an unexplained regenerative anemia and mild hepatopathy. **Methods:** Clinicopathological evaluations, urinary mass spectroscopy, and molecular genetic studies were performed. Urine metabolic screening identified increased biliverdin concentrations in both cases relative to control dogs. **Results:** Whole genome and Sanger sequencing revealed that each case was homozygous for large deletions in *BLVRA*: UU_Cfam_GSD_1.0/canFam4 chr18:6,532,022–6,551,313 (19,292 bp) in Case 1 and chr18:6,543,863–6,545,908 (2046 bp) in Case 2. These variants were predicted to result in major *BLVRA* truncations (ENSCAFT00805017018.1 p.[Lys117-Lys296del] and p.[Ala154fs], respectively) and loss of enzyme function. In a genomic variant database, 671 dogs from 63 breeds had coverage over these regions, ruling out homozygosity for the *BLVRA* deletions. A gene defect for the regenerative anemia in Case 1 was not discovered. **Conclusions:** While expected to be rare, genotyping for the *BLVRA* deletions can be used to identify other affected and carrier dogs. This study illustrates the use of targeted metabolic and genomic screening as key diagnostic tools to diagnose a rare metabolic disorder. These are the first confirmed cases of biliverdinuria caused by *BLVRA* defects in non-human mammals.

## 1. Introduction

Green urine is rare in mammals. When it does occur, it is typically transient and acquired from food coloring, medications (e.g., propofol, amitriptyline, methylene blue), or *Pseudomonas* urinary tract infections [1,2,3,4,5,6]. Persistent or recurrent green urine is extraordinarily rare and is consistent with biliverdinuria from an inborn error in heme catabolism. Reports of biliverdinuria in domestic animals are lacking.

The first step in heme degradation is catabolism by heme oxygenase to produce biliverdin, iron, and carbon monoxide [7]. In mammals, biliverdin is rapidly reduced to bilirubin by biliverdin reductase. There are two biliverdin reductase isoenzymes, A and B, that are encoded by the genes *BLVRA* and *BLVRB*, respectively. Biliverdin reductase A has ubiquitous expression and is the predominant isoenzyme in the adult, while biliverdin reductase B appears to only reduce biliverdin in the fetus and neonate [7]. In healthy mammals, serum and plasma concentrations of biliverdin are very low to undetectable because biliverdin reductase A efficiently reduces biliverdin to bilirubin [8,9,10].

Only two rare variants in *BLVRA* have been reported to cause hyperbiliverdinemia and biliverdinuria in case reports of humans presenting with “green jaundice” and cirrhosis or obstructive cholestasis [10,11]. Skin and bodily fluids, including plasma, serum, urine, and milk (in the case of a lactating woman), had a greenish appearance [10,11]. These discolorations were the primary clinical phenotype of the *BLVRA* deficiency. Choleliths were also diagnosed in three affected women [11], but it was not possible to determine whether the hyperbiliverdinemia contributed to their formation. Other long-term health effects of *BLVRA* deficiency have not been described.

The aim of this study was to characterize the clinical and metabolic phenotypes of two dogs with life-long green urine to diagnose, for the first time, biliverdinuria and the underlying pathogenic *BLVRA* variants in non-human mammals.

## 2. Materials and Methods

### 2.1. Case Identification, Ethics Statement, and Data Collection

The two cases were presented to Colorado State University Veterinary Teaching Hospital (CSU VTH) and VCA West Los Angeles Animal Hospital (VCA West LA), respectively, for the evaluation of green urine since puppyhood. Data were obtained from medical records, including the results of serum chemistry panels, complete blood counts, urinalyses, abdominal imaging, and other clinical diagnostic tests. Informed owner consent was obtained for genetic research under a protocol approved by the University of Minnesota (UMN) Institutional Animal Care and Use Committee (IACUC, protocol 2201-39732A). Whole blood was collected from each case in an ethylenediaminetetraacetic acid tube and a Tempus Blood RNA Tube (Thermo Fisher Scientific, Waltham, MA, USA) for DNA and RNA, respectively. Urine was collected, placed in a light-protected bag or container, and frozen at −20 °C or −80 °C. Samples were shipped overnight with a cold pack to the UMN for analysis.

### 2.2. Targeted Metabolomic Analyses

Upon arrival at the UMN, urine samples were protected from light and stored at −80 °C until analysis. For each case, biobanked urine samples were selected from two control dogs that were age- (within two years) and sex-matched to the case. Control samples had been collected from dogs as part of ongoing studies on urolithiasis with informed owner consent and UMN IACUC approval (protocol 2107-39264A). All four controls had yellow-colored urine. Two of the controls were healthy, and two had a history of calcium oxalate uroliths.

Ten µL of conjugate bilirubin (50 ng/µL) and 50 µL of bilirubin-D4 (10 ng/µL) were added to 10 µL of urine from each dog. Sample extraction was performed with 300 µL of 1% formic acid in acetonitrile. Samples were vortexed for ten minutes at 13,000 rpm and centrifuged for ten minutes at 13,300 rpm. A 100 µL aliquot of the supernatant was transferred to a loading vial and subsequently diluted at a 1:1 ratio with mobile phase A (1/1; *v*/*v*, sample/10 mM ammonium formate, 0.125% formic acid). Samples were analyzed via liquid chromatography-tandem mass spectrometry (LC-MS/MS) utilizing multiple reaction monitoring.

Bilirubin and biliverdin LC-MS/MS analysis was conducted using a Waters Acquity Premiere UHPLC (Waters Corp, Milford, MA, USA) coupled to a Sciex QTRAP 6500 (SCIEX Corp, Framingham, MA, USA). Chromatographic separation was performed with an Acquity Premier BEH C18 UPLC column (2.1 × 50 mm, 1.7 µM) at 45 °C. Mobile phases of separation consisted of A: 10 mM ammonium formate, 0.125% formic acid, and B: acetonitrile. Positive ion mode was utilized for acquisition, with an injection volume of 10 µL. The elution started with a linear gradient of 0–5 min from 60–0% A; 5–7 min isocratic hold at 0% A; 7–7.10 min from 0–60% A; and 7.10–8 min isocratic hold at 60% A, with a consistent flow rate of 0.5 mL/min. The acquired data were imported into Skyline software (v. 23.1.0.380; RRID SCR_014080) for analysis.

Results are reported as metabolite concentrations (µg/dL) and metabolite-to-creatinine ratios (µg/mg); for the latter, urine creatinine concentrations were measured using a modified Jaffe procedure (Beckman Coulter AU480, Brea, CA, USA). The median of the triplicates was calculated for each case and for all controls combined.

### 2.3. Genetic Analyses

Genomic DNA was extracted from whole blood samples using the Gentra Puregene Blood Kit (Qiagen Sciences, Germantown, MD, USA). For Case 1, library preparation and whole genome sequencing (WGS) was performed by Neogen GeneSeek Operations (Neogen Corporation, Lansing, MI, USA). Libraries were generated using the Illumina DNA Prep Kit and sequenced using an Illumina NovaSeq 6000 system with 150 bp paired-end reads for an average coverage of 33x (Illumina, San Diego, CA, USA). Data were processed using a standardized pipeline modified from the Genome Analysis Toolkit (GATK) Best Practice guidelines [12,13]. Briefly, after quality control measures, reads were mapped to the UU_Cfam_GSD_1.0/canFam4.0 dog reference assembly (GenBank accession GCA_011100685.1) with the Burrows-Wheeler Alignment tool [14], variants were called using GATK HaplotypeCaller, and annotations were performed using Ensembl’s Variant Effect Predictor (VEP; RRID SCR_007931) [15]. Variants identified in Case 1 were filtered against an internal database of whole genome variant calls from 671 dogs of 62 breeds for those that were unique to the case (allele frequency, af = 0 in database) using the OnlyWAG pipeline [12]. For variants present in a homozygous state in Case 1, rare variants (af < 0.005 in database) were also extracted. The variant list was screened for those present in the candidate gene for biliverdinuria, *BLVRA*, and reads across *BLVRA* were visually inspected using Integrative Genomics Viewer (IGV; RRID SCR_011793) [16]. To assess the extent of homozygosity around *BLVRA*, variant calls were evaluated for the full length of chromosome 18.

Due to an additional phenotype of a regenerative anemia in Case 1, filtering steps were performed to evaluate for potential causal variants for this phenotype. First, the variants were filtered to identify those with a VEP impact of “moderate” (e.g., missense variants) or “high” (e.g., stop gained, frameshift, splice site variants). The moderate-to-high impact variant list was next screened for those present in a list of candidate genes for the phenotype of “anemia” as generated by Phenolyzer (http://phenolyzer.wglab.org, accessed on 23 August 2024) using a score threshold of ≥0.05 [17]. Clinical phenotypes associated with genes were retrieved from the Online Mendelian Inheritance in Man (OMIM, omim.org, accessed on 9 September 2024; RRID SCR_006437) [18]. The pathogenicity of missense variants was assessed using two programs: MutPred2 (RRID SCR_010778) and the HumVar model of PolyPhen-2 (RRID SCR_013189) [19,20].

For Case 2, a different approach was used that did not include WGS. Case 2 was genotyped for a large *BLVRA* deletion identified in Case 1 using a PCR-based assay with two experiments (Table S1). In the first, the primers were located within the deleted region. In the second, the primers flanked the deletion. Homozygosity for the reference sequence resulted in a PCR product only for the first, and homozygosity for the deletion resulted in a PCR product only in the second; heterozygosity would be expected to result in PCR products in both experiments. After confirming that Case 2 did not share the same variant, PCR and Sanger sequencing were performed for all *BLVRA* exons (transcript ENSCAFT00805017018.1). When a large deletion was suspected, long-range PCR and primer walking were used to determine its breakpoints. The primers and conditions for each PCR are provided in Tables S2 and S3. Primer3Plus (RRID SCR_003081) was used for primer design [21].

Copy number variants, such as large deletions, often originate from non-allelic homologous recombination in dogs [22]. Therefore, to investigate for potential mechanisms that caused the case deletions, the sequence up- and downstream of the breakpoints for each *BLVRA* deletion was evaluated using the RepeatMasker (RRID SCR_012954) track in the University of California Santa Cruz Genome Browser (RRID SCR_005780) [23,24]. This track shows the location of repetitive elements, including short interspersed nuclear elements (SINEs), long interspersed nuclear elements (LINEs), long terminal repeat elements (LTRs), DNA repeat elements, simple repeats (microsatellites), low complexity repeats, satellite repeats, RNA repeats, and other repeats (e.g., rolling circle).

The internal WGS variant call database of 671 dogs of 63 breeds was used to determine the frequency of homozygosity for the *BLVRA* deletions in the broader dog population. Due to the large size of the deletions, these variants were not called in the WGS pipeline. Therefore, the frequency of homozygosity was determined by evaluating read numbers over the deleted regions. For each dog, a read depth average of five or greater across the region was considered sufficient to rule out homozygosity for the deletion.

InterPro (RRID SCR_006695, http://www.ebi.ac.uk/interpro, accessed on 4 August 2024) was used to identify domains in the entry for the human *BLVRA* protein (UniProt A0A140VJF4) [25]. UniProt was used to determine the location of binding sites (RRID SCR_002380, uniport.org, accessed on 9 September 2024) [26].

### 2.4. RNA Analyses

Whole blood RNA was extracted from the case samples collected in a Tempus Blood RNA Tube (Thermo Fisher Scientific, Waltham, MA, USA) using the Tempus Spin RNA Isolation Reagent Kit (Thermo Fisher Scientific, Wilmington, DE, USA). Whole blood RNA was also selected from five control dogs; these samples were collected and stored using identical methodology and had been biobanked under the same UMN IACUC protocol as the cases (protocol 2201-39732A). The RNA quantity and quality were evaluated using a Nanodrop 8000 (Thermo Fisher Scientific, Wilmington, DE, USA). Synthesis of cDNA was achieved using 500 ng of total RNA and the iScript Reverse Transcription Supermix (BioRad Laboratories, Hercules, CA, USA).

Primer3Plus (RRID SCR_003081) was again used for *BLVRA* primer design [21]. For Case 2, cDNA sequencing was performed using primers spanning *BLVRA* exon 6 to confirm exon skipping (Table S4). For both cases, a quantitative real-time PCR (QT-rPCR) was performed to determine *BLVRA* expression. The QT-rPCR primers were within exons 3 and 4 and, thus, not within the deleted regions for either case (Table S4). Three housekeeping genes for canine whole blood, *GUSB*, *HNRNPH1*, and *CG14980*, were used for normalization with previously published primers [27,28]. The QT-rPCR reactions were run in triplicates for each sample with iTaq Universal SYBR Green Supermix (Bio-Rad Laboratories, Hercules, CA, USA) with optimization for the Tm and an annealing temperature of 56 °C on a CFX96 Touch Real-Time PCR Detection System (RRID SCR_018064; BioRad Laboratories, Hercules, CA, USA). Amplification efficiency from standard curves was calculated as 106.2–116.9% by the CFX Maestro Software 2.3.

## 3. Results

### 3.1. Clinical Case Descriptions

**Figure 1:**
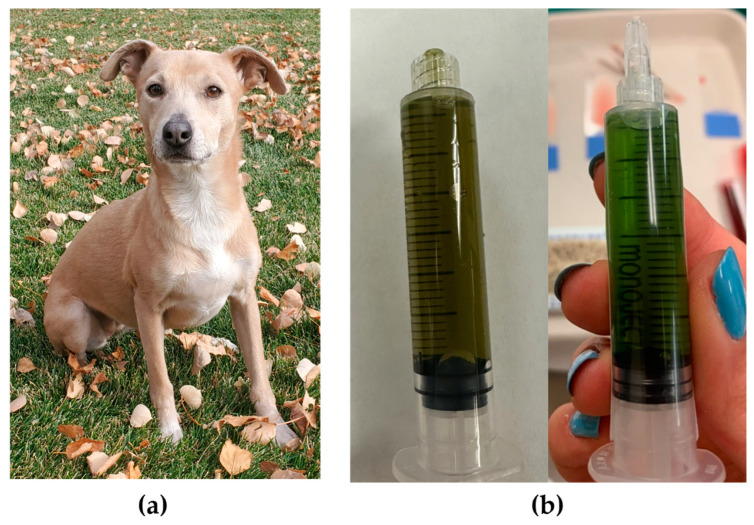
Images showing the (**a**) physical appearance and (**b**) urine (from two dates) for Case 1, a mixed-breed dog with green urine.

Case 1 was a three-year-old female spayed mixed-breed dog weighing 10.1 kg (Figure 1a). The dog was referred to the Internal Medicine Department at CSU VTH for evaluation of grossly green urine (Figure 1b) and a regenerative anemia that were both first noted at four months of age. Family history was unknown. Breed ancestry testing through the Wisdom Panel (Mars Petcare, Franklin, TN) reported the following top four breeds: Border Collie (26%), American Staffordshire Terrier (19%), Labrador Retriever (16%), and American Pit Bull Terrier (11%).

Abnormalities identified in earlier diagnostic test results included persistently green urine on urinalysis (no bacterial growth on urine culture), a persistent macrocytic hypochromic regenerative anemia with a hematocrit of 25–33% (reference interval 37–62%) on complete blood count analysis, and increased serum gamma-glutamyltransferase (GGT) activity at 4–40 IU/L (reference interval 0–11 IU/L). An abdominal ultrasound performed while the dog was receiving prednisone therapy (see Table S5 for all therapies) revealed hyperechoic hepatic parenchyma with bilaterally small adrenal glands. A cytologic evaluation of hepatic aspirate samples showed hepatocellular vacuolization consistent with glycogen or hydropic change, atypical pigment accumulation consistent with increased iron and cholestasis, mononuclear inflammation, and mild extramedullary hematopoiesis. A cytologic evaluation of splenic aspirates showed reactive lymphoid tissue with marked extramedullary hematopoiesis. Serum iron was measured and was mildly increased at 311 μg/dL (reference interval 77–253 μg/dL). An infectious disease PCR panel was negative for all species tested (*Babesia* spp., *Anaplasma* spp., *Ehrlichia* spp., *Rickettsia* spp., *Hepatozoon* spp., *Leishmania* spp., *Neorickettsia* spp., *Bartonella* spp., *Mycoplasma haemocanis*, *Candidatus Mycoplasma haematoparvum*). The dog tested clear of two genetic variants previously reported as causes of phosphofructokinase deficiency (OMIA:000421-9615) [29,30]. The dog had received therapy with antimicrobials, immunosuppressives, and other medications (Table S5), which failed to resolve the green urine and anemia.

**Figure 2:**
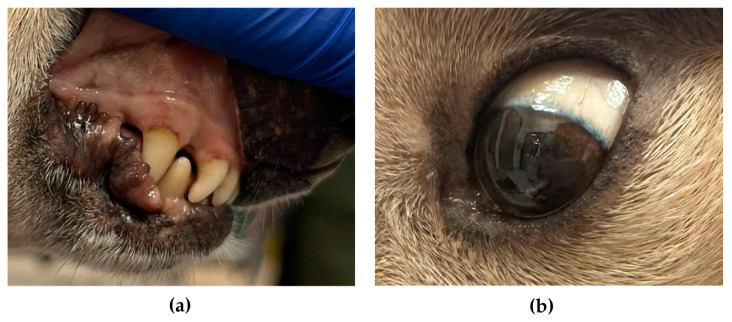
Mucous membrane (**a**) and sclera (**b**) of Case 1 showed a slight greyish-tan discoloration.

On presentation to CSU VTH, a physical examination revealed a slight grey-tan discoloration of the mucus membranes and sclera (Figure 2), light brown feces with mild green tint, and mild diffuse muscle atrophy. Multiple diagnostics were performed at this initial evaluation and at two follow-up visits spanning a 9-month period. Urinalyses showed green discoloration with dipstick proteinuria (1+ to 2+) and bilirubinuria (1+ to 2+) (Table S6). The results of complete blood counts and serum chemistry profiles were consistent with those obtained at the primary veterinary clinic with a macrocytic hypochromic regenerative anemia (hematocrit of 25–32%, reference interval 40–55%; Table S7); neither spherocytes nor in-saline agglutination were noted. Serum bilirubin concentration was within the reference interval (0.1 mg/dL, reference interval 0.0–0.2 mg/dL), but GGT was persistently elevated (16–23 IU/L, reference interval 0–9 IU/L; Table S7). Other mild abnormalities consistent with corticosteroid therapy (thrombocytosis, neutrophilia, monocytosis, and increases in serum glucose concentration and alkaline phosphatase activity) were noted; these had not been present prior to prednisolone therapy. Pre- and post-prandial serum bile acids concentrations were within the laboratory reference interval.

**Figure 3:**
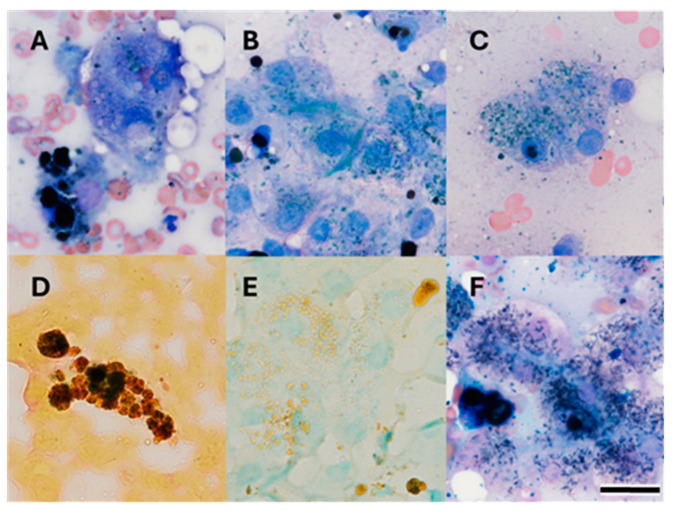
Photomicrograph of hepatic aspirates from Case 1 showing marked pigment accumulation. (**A**–**C**) Wright-Giemsa stain. (**D**)—Hall’s bilirubin. (**E**)—Rhodanine stain. (**F**)—Prussian Blue. Bar = 20 μm.

An abdominal ultrasound at CSU VTH showed a normal biliary system (no cholelithiasis detected), diffusely hyperechoic liver with multiple hypoechoic nodules, mild reticulonodular splenic pattern with few variable sized hypoechoic nodules, and mildly small adrenal glands. A cytologic evaluation of the liver revealed mild hepatocellular rarefaction, mild lymphocytic inflammation, and a marked accumulation of pigment with multiple morphologies including brown-black globular, blue-black acicular, and amorphous green-blue forms, (Figure 3A–C). Some of the pigment was positive with Hall’s bilirubin, suggesting bilirubin (Figure 3D). Copper was not documented by rhodanine stain, but some of the intracellular and extracellular pigment was positive with Prussian blue (Figure 3F). Splenic cytology showed a marked accumulation of iron and extramedullary hematopoiesis.

Based upon the persistently green urine and no identified external triggers, a hereditary biliverdinuria was suspected. It was unclear if the anemia and hepatic changes were related or caused by a hereditary erythrocyte defect.

**Figure 4:**
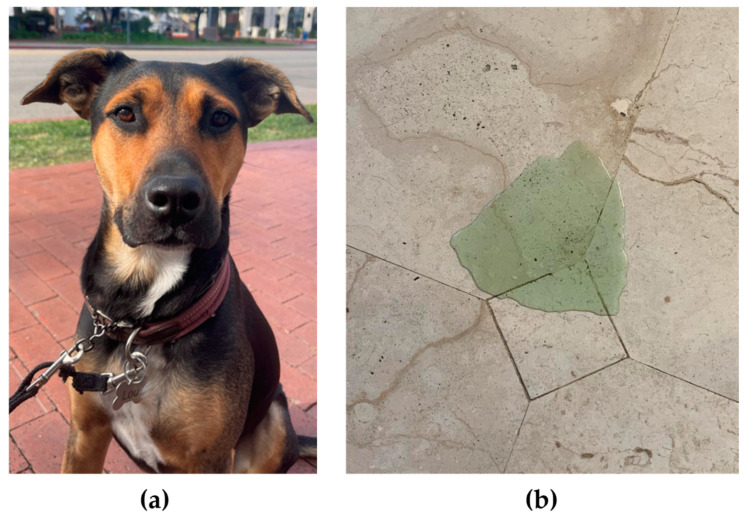
Images showing the (**a**) physical appearance and (**b**) urine for Case 2, a mixed-breed dog with green urine.

Case 2 was a three-year-old male neutered mixed-breed dog weighing 20.6 kg (Figure 4a). The dog presented to the Internal Medicine Department at VCA West LA for evaluation of life-long grossly green urine (Figure 4b) and feces. Family history was unknown, and no breed ancestry studies were performed.

Multiple previous urinalyses showed color ranging from yellow to green with intermittent dipstick protein (negative to trace); urine was consistently negative for dipstick bilirubin (Table S6). Urine culture showed no bacterial growth. A Fecal Dx Profile with *Giardia* (Idexx Laboratories, Westbrook, ME, USA) was negative for ova and parasites and for *Giardia*, hookworm, whipworm, and roundworm antigens. Multiple complete blood counts were normal with a hematocrit of 53–58% (laboratory reference interval of 36–60%), and serum chemistry panels also showed values within reference intervals (Table S7), including bilirubin at 0.1 mg/dL (reference interval 0.0–0.3 mg/dL). Serum concentrations of pre- and post-prandial bile acids, thyroxine, cobalamin, folate, and Spec cPL (Idexx Laboratories, Westbrook, ME, USA) were within reference intervals. Trypsin-like immunoreactivity was increased at >50 µg/L (reference interval 5–35 µg/L). Abdominal radiographs had been performed once after acute dietary indiscretion, and no abnormalities were identified according to the radiologist report. No specific therapy had been administered for the treatment of the green urine or feces.

On presentation to VCA West LA, a physical examination was normal. The dog tested negative for heartworm antigen and *Borrelia burgdorferi*, *Ehrlichia* spp., and *Anaplasma phagocytophilum* antibodies. Again because of persistent green urine with no identified triggers, hereditary biliverdinuria was suspected.

### 3.2. Targeted Metabolomic Analyses

Targeted metabolomics were performed to quantify biliverdin and bilirubin concentrations in urine samples from both cases and age- (within two years) and sex-matched controls. The urine samples for metabolomics were collected from Case 1 at 4.5 years and Case 2 at 3.9 years; the sample analyzed from Case 1 was grossly green, but the sample from Case 2 was yellow in appearance (Figure S1).

Urinary biliverdin and bilirubin data are reported in Table 1. Urinary biliverdin/creatinine ratios were 43-fold and 10-fold greater in Case 1 and Case 2, respectively, than controls. In contrast, urinary bilirubin/creatinine ratios overlapped between the cases and controls. Intensity plots for individual samples are shown in Figure S2.

### 3.3. BLVRA Genetic Analyses

**Figure 5:**
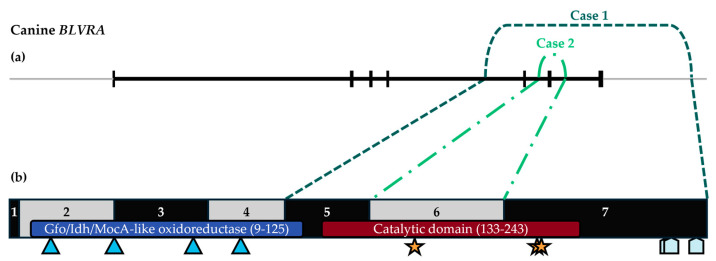
Schematic of Case 1 and Case 2 deletions in the canine biliverdin reductase gene and protein. (**a**) *BLVRA* (ENSCAFT00805017018.1) is shown in black with vertical bands representing exons and borders of Case 1 and Case 2 deletions shown in dark green (simple dashed) and light green (dot and dashed), respectively; the light grey line represents up- and downstream intergenic sequence. Exon 6 skipping in Case 2 was confirmed through mRNA analysis. (**b**) *BLVRA* protein (UniProt A0A8C0QEH9) is shown with the locations of the Gfo/Idh/MocA-like oxidoreductase and catalytic domains, NADP^+^ binding sites (blue triangles), residues forming the carboxylate pocket (gold stars), and Zn^2+^ binding sites (light blue pentagons).

The results of WGS for Case 1 were analyzed for *BLVRA* variants. The genomic variant calling pipeline did not identify any unique or rare *BLVRA* variants in Case 1. However, a visual inspection of reads over *BLVRA* revealed homozygosity for a 19,292 bp deletion at chr18:6,532,022–6,551,313 (UU_Cfam_GSD_1.0/canFam4) that spanned *BLVRA* exons 5–7 (transcript ENSCAFT00805017018.1). This variant was predicted to result in the truncated mRNA product p.[Lys117-Lys296del]. The deleted exons spanned the entire biliverdin reductase catalytic domain (IPR015249; Figure 5), and, thus, the deletion is predicted to result in loss of enzyme activity. An analysis of variant calls across chromosome 18 revealed that the Case 1 deletion fell within a large run of homozygosity that encompassed 46.9 Mb (chr18:173,323–47,043,187). Both the upstream and downstream sequences surrounding the deletion breakpoints fell within LINEs (L1 type).

A PCR-based assay was developed to genotype for the 19.3 kb deletion present in Case 1. Case 2 tested clear of the deletion. Exonic sequencing of *BLVRA* was performed, and exon 6 consistently would not amplify. A long-range PCR with primer walking was performed and identified a 2046 bp deletion at chr18:6,543,863–6,545,908 (UU_Cfam_GSD_1.0/canFam4) that spanned *BLVRA* exon 6 in its entirety. This variant was predicted to result in exon-skipping with disruption of the reading frame and production of a premature stop codon after 12 amino acids in the new C-terminal sequence, p.[Ala154ValfsTer12], resulting in loss of greater than 50% of the catalytic domain (Figure 5). An analysis of the upstream and downstream sequences surrounding the Case 2 deletion breakpoints did not identify any repetitive elements.

All 671 dogs in the internal WGS variant database had coverage over *BLVRA* exons 5, 6, and 7, ruling out homozygosity for either deletion; direct genotyping for the deletions was not performed in these database dogs.

### 3.4. BLVRA RNA Analyses

The Case 2 *BLVRA* deletion was confirmed to result in exon 6 skipping through analysis of cDNA sequencing of peripheral blood RNA (Figure 5). The effects of the deletions on *BLVRA* mRNA expression in blood were measured with QT-rPCR using primers placed in undeleted exons. The results were determined for each case and for five control dogs. The relative normalized expression of *BLVRA* mRNA was 0.8 for Case 1, 1.4 for Case 2, and 1.0 (0.6–1.6) for the controls.

### 3.5. Case 1 Analyses of Candidate Variants for Anemia

Approximately 73 homozygous variants (31 unique and 42 rare) and 60 heterozygous variants (all unique, as per filtering methods) were identified in Case 1 (Table S8); 42 of these variants had a Phenolyzer score ≥0.05 related to anemia and were scrutinized further (Table S9). Of those, 3 were high impact (2 premature stop codons and 1 frameshift), and 16 were missense variants predicted to be pathogenic by at least one of the two prediction programs; the other 23 variants were missense variants with benign pathogenicity predictions. Based on OMIM clinical phenotypes, none of the 19 variants resided in genes considered likely candidates for the regenerative anemia present in Case 1. Further evaluation for putative causal variants for anemia was not performed.

## 4. Discussion

Clinicopathological, metabolic, and molecular investigations of two unrelated adult mixed dogs in the United States document the first cases of hereditary biliverdinuria in non-human mammals. Both cases were homozygous for different large exonic *BLVRA* deletions. This study greatly expands our knowledge about this extraordinarily rare inborn error of metabolism and might help with the clinical recognition and diagnosis of future cases in dogs, humans, and other mammals.

The canine (UniProt A0A8C0QEH9) and human (UniProt P53004) *BLVRA* proteins are highly homologous; both are 296 amino acids in length, and they have 94% sequence identity. The *BLVRA* deletions identified in the two dogs with biliverdinuria overlapped with both spanning exon 6. According to InterPro predictions, the deletions result in the complete or partial loss of the *BLVRA* catalytic domain. The severely truncated mutant proteins would, thus, be unable to reduce biliverdin to bilirubin. Of note, the current InterPro prediction for the catalytic domain differs from the past modeling that predicted its location at the N terminus [31,32]. An alternative explanation for the deleterious effects of the deletions is loss of substrate binding capacity. Three of the residues deleted, Arg172, Arg225, and Arg227, are predicted to form a “carboxylate pocket” that stabilizes substrate binding [31]. Given that the deletion in Case 2 resulted in a frameshift and premature stop codon, decreased mRNA expression from nonsense-mediated decay is another explanation for the deleterious effect of the variant [33]. However, this was not supported by the QT-rPCR results, which showed similar mRNA expression levels in the cases and healthy controls. In any case, the functional impact of the variants is likely loss of enzyme function. A limitation of this study is that *BLVRA* enzyme activity studies were not directly performed to confirm loss of function. The only two *BLVRA* variants reported in humans with biliverdinuria resulted in premature stop codons, p.Arg18* and p.Ser44*, and the latter was confirmed to lack biliverdin reductase activity in an in vitro system [10,11].

In each of the dogs with biliverdinuria, their respective *BLVRA* deletions were identified in a homozygous state, consistent with an autosomal recessive inheritance for the inborn error of metabolism. In one of the previous reports of biliverdinuria in human patients, the causal *BLVRA* variant was also present in a homozygous state [11]. Yet, in another incompletely characterized patient (without enzyme activity studies), only a heterozygous *BLVRA* variant was detected [10]. A complicating factor in determining inheritance of *BLVRA* deficiency is that it appears to have incomplete penetrance for hyperbiliverdinemia in the absence of cholestatic disease [10,11]. In the present study, we were unable to assess whether any healthy dogs were heterozygous for either of the *BLVRA* deletions detected in the two biliverdinuria cases. The cases were both privately owned mixed-breed dogs, and their family members were unknown. All 671 dogs in our internal WGS variant call database had coverage over *BLVRA* exons 5–7, but no large genotyping screening was performed. While this rules out homozygosity for both deletions, heterozygosity calling was not attempted due to variable read depth per dog.

The deletion in Case 1 resided within a long run of homozygosity (46.9 Mb). The extent of homozygosity within a population is related to inbreeding, and longer runs of homozygosity are present in purebred dogs relative to village dogs [34]. The average length of runs of homozygosity varies between breeds with estimates of 1.0 Mb (range 0.5–7.8 Mb) in the Labrador Retriever, 2.3 Mb (range 0.3–51.4 Mb) in the Bernese Mountain Dog, and 5.9 Mb (range 1.0–90.2 Mb) in the Leonberger [35,36,37]. The greater than average length of the run of homozygosity on chromosome 18 in Case 1 likely indicates recent inbreeding. Since family history was not available, we were not able to determine whether Case 1 was the result of the breeding of first-degree relatives. As we did not analyze the extent of homozygosity across the genome, we also cannot rule out partial/segmental isodisomy, a form of uniparental disomy, as the source [38]. Either mechanism could explain why such a unique deletion was present in a homozygous state in Case 1. Since a candidate gene approach was used for Case 2, it was not possible to estimate the extent of homozygosity surrounding the dog’s deletion.

An evaluation for repetitive elements revealed that the Case 1 deletion breakpoints occurred within LINEs. Greater than 96% of copy number variants in the dog genome have breakpoints residing within LINEs [22]. This supports non-allelic homologous recombination as the origin of the Case 1 deletion. No repetitive elements were detected in the sequence surrounding the deletion breakpoints for Case 2, and the origin of this deletion remains undetermined.

In this study, we used LC-MS/MS to quantify urinary biliverdin and bilirubin. While both cases had biliverdinuria relative to matched controls, the urinary biliverdin concentration was considerably greater in Case 1 than Case 2 even with normalization to creatinine concentrations. Given the regenerative anemia in Case 1, one possibility is that this dog had increased red blood cell turnover, resulting in higher levels of heme degradation. A limitation in interpreting the degree of relative biliverdinuria is the lack of repeated analyses and information about day-to-day variability. While Case 2 had reports of green urine (and feces) throughout its life, its urine was intermittently yellow, including the sample submitted for metabolomic analyses. Metabolomic analyses of additional samples are needed to confirm the degree of biliverdinuria in each case.

The only clinical sign of *BLVRA* deficiency in these dogs was green urine (and feces). In *BLVRA* knockout mice, green discoloration of the bile and gallbladder is the major phenotype [39]. Although markers of oxidative stress are increased, major pathologic changes are not described [39]. In the two reports of human patients with *BLVRA* deficiency, green discoloration of skin, sclera, and bodily fluids was only noticed in the presence of cholestatic disease [10,11]. In one patient, the cholestasis might have been caused by alcoholic cirrhosis. Other patients had choleliths, but it is unknown if their *BLVRA* deficiency contributed to their formation because cholelithiasis is common and other risk factors were present (e.g., female gender, pregnancy, and obesity) [11,40]. Hyperbiliverdinemia can resolve post-resolution of cholestasis in human patients with BLRVA deficiency, but biliverdin concentrations in urine, milk, and bile remain elevated [11]. Though one of the dogs presented here had a persistent mild increase in GGT activity and imaging and cytological changes consistent with a mild hepatopathy, there was no evidence of cholestasis and cholelithiasis. The other dog did not show any hepatic abnormalities.

While Case 2 was healthy aside from the biliverdinuria, Case 1 exhibited a regenerative anemia that persisted for the entire observation period of close to two years. Case 1 had no evidence of blood loss or underlying diseases or triggers for anemia. While serum bilirubin concentrations were normal in Case 1, the *BLVRA* deficiency could mask elevations due to the lack of reduction of biliverdin to bilirubin. The pathologic findings in the liver might be consistent with iron accumulation from increased red cell (and, therefore, heme) turn-over. Slight greyish-tan discoloration of the mucous membranes and sclera was noted in Case 1. This might occur with biliverdin pigment accumulation in the tissues, but serum from the patient was grossly normal in color; serum and plasma concentrations of biliverdin were not quantified. Screening for known inherited and acquired causes of hemolytic anemia in dogs did not reveal a cause in Case 1. Anemia and hemolysis are not features of *BLVRA* knockout mice and were also not described in the few human patients with *BLVRA* deficiency [10,11,39]. The WGS of Case 1 was analyzed, but we did not identify a putative causal variant for a hereditary erythrocyte disorder. While 133 variants passed the filtering based on absence or rarity in an internal WGS database and impact on the protein (e.g., nonsense, missense), Phenolyzer scores for gene association with anemia were low, and no variant resided in a gene reported to cause a regenerative anemia. Thus, the cause of the apparent hemolytic anemia in Case 1 remains unexplained.

## 5. Conclusions

In conclusion, this is the first report of hereditary biliverdinuria and *BLVRA* variants in non-human mammals. The clinicopathological and metabolic analyses suggest that this can be a benign disorder with green urine as the predominant clinical sign. The molecular characterizations highlight exon 6 as a critical region of the protein, consistent with predictions that the catalytic domain spans this exon. Genotyping for the *BLVRA* deletions might help identify other affected and carrier dogs, but there is no indication that either *BLVRA* deletion would be common in a certain breed or dog population. This study also illustrates the use of targeted metabolic and genomic screening as key diagnostic tools. This information greatly adds to the understanding of an extremely rare inborn error of metabolism in heme catabolism and might help with the recognition, diagnosis, and management of future cases of hereditary biliverdinuria across species.

## Figures and Tables

**Table 1:** Urine biliverdin and bilirubin in Case 1, Case 2, and matched controls measured by liquid chromatography-tandem mass spectrometry with and without normalization to urinary creatinine concentration.

Metabolite	Case 1	Case 2	Controls, *n* = 4 Median (Range)
Biliverdin (µg/dL)	22.1	4.0	0.3 (0.1–0.8)
Biliverdin/creatinine (µg/mg)	0.130	0.031	0.003 (0.001–0.005)
Bilirubin (µg/dL)	0.5	2.2	1.3 (0.4–20.0)
Bilirubin/creatinine (µg/mg)	0.003	0.017	0.030 (0.003–0.110)

## Data Availability

The WGS data generated in this study are available in NCBI’s Short Read Archive under BioProject PRJNA937381 with the accession number SRR30832994. The raw metabolomics files will be made available upon request to the corresponding author.

## Supplementary Materials

The following supporting information can be downloaded at https://www.mdpi.com/article/10.3390/genes15121561/s1: Table S1. Primers for genotyping of the multiexonic *BLVRA* deletion (chr18:6,532,022–6,551,313del, UU_Cfam_GSD_1.0/canFam4) detected in Case 1. Table S2: Primers for *BLVRA* (ENSCAFT00805017018.1) exons. Table S3. Primers for the exonic *BLVRA* deletion (chr18:6,543,863–6,545,908del, UU_Cfam_GSD_1.0/canFam4) detected in Case 2. Table S4: Primers for cDNA sequencing and QT-rPCR. Table S5: Case 1 therapies administered with age of the dog at the time of therapy, dosage, route, and duration. Table S6: Urinalysis results of Case 1 and Case 2. Table S7: Complete blood count and serum chemistry panel results for Case 1 and Case 2. Table S8: Genomic variants identified through whole genome sequencing (WGS) of Case 1 that passed preliminary filtering criteria. Table S9: Forty-two variants identified in whole genome sequencing of Case 1 that met filtering criteria, including involving a gene with a Phenolyzer score for “anemia” of ≥0.05. Figure S1: Urine samples from Case 1 (a) and Case 2 (b) used for analysis of biliverdin and bilirubin concentrations. Figure S2: Plots of intensity versus retention time (minutes) for biliverdin (“verdin”) and bilirubin (“rubin”) measured by liquid chromatography-tandem mass spectrometry in urine from Case 1 (a), Case 2 (b), and control dogs (c–f). Note that the y-axis limit for Case 1 is 30-fold greater than all other plots.

## References

[1] Flaherty D., Auckburally A. (2017). Green Discolouration of Urine Following Propofol Infusion in a Dog. J. Small Anim. Pract..

[2] Lasica A., Cortesi C., Milani G.P., Bianchetti M.G., Schera F.M., Camozzi P., Lava S.A.G. (2023). Propofol-Associated Urine Discoloration: Systematic Literature Review. Pharmacology.

[3] Moussa M., Chakra M.A., Papatsoris A.G., Dellis A. (2022). Green Urine Due to Pseudomonas Urinary Tract Infection: An Unusual Occurence. Am. J. Emerg. Med..

[4] Greenberg M. (2008). Verdoglobinuria. Clin. Toxicol..

[5] Koratala A., Leghrouz M. (2017). Green Urine. Clin. Case Rep..

[6] Stone H.H., Martin J.D., Graber C.D. (1964). Verdoglobinuria: An Ominous Sign of Pseudomonas Septicemia in Burns. Ann. Surg..

[7] Weaver L., Hamoud A., Stec D.E., Hinds T.D. (2018). Biliverdin Reductase and Bilirubin in Hepatic Disease. Am. J. Physiol.-Gastrointest. Liver Physiol..

[8] Larson E.A., Evans G.T., Watson C.J. (1947). A Study of the Serum Biliverdin Concentration in Various Types of Jaundice. J. Lab. Clin. Med..

[9] Garner R.J. (1953). Bile Pigment Metabolism in Cattle. J. Comp. Pathol. Ther..

[10] Gåfvels M., Holmström P., Somell A., Sjövall F., Svensson J., Ståhle L., Broomé U., Stål P. (2009). A Novel Mutation in the Biliverdin reductase-A Gene Combined with Liver Cirrhosis Results in Hyperbiliverdinaemia (Green Jaundice). Liver Int..

[11] Nytofte N.S., Serrano M.A., Monte M.J., Gonzalez-Sanchez E., Tumer Z., Ladefoged K., Briz O., Marin J.J.G. (2011). A Homozygous Nonsense Mutation (c.214C->A) in the Biliverdin Reductase α Gene (BLVRA) Results in Accumulation of Biliverdin during Episodes of Cholestasis. J. Med. Genet..

[12] Cullen J.N., Friedenberg S.G. (2023). Whole Animal Genome Sequencing: User-Friendly, Rapid, Containerized Pipelines for Processing, Variant Discovery, and Annotation of Short-Read Whole Genome Sequencing Data. G3 Genes Genomes Genet..

[13] Van der Auwera G.A., O’Connor B.D. (2020). Genomics in the Cloud: Using Docker, GATK, and WDL in Terra.

[14] Li H., Durbin R. (2009). Fast and Accurate Short Read Alignment with Burrows–Wheeler Transform. Bioinformatics.

[15] McLaren W., Gil L., Hunt S.E., Riat H.S., Ritchie G.R.S., Thormann A., Flicek P., Cunningham F. (2016). The Ensembl Variant Effect Predictor. Genome Biol..

[16] Robinson J.T., Thorvaldsdóttir H., Winckler W., Guttman M., Lander E.S., Getz G., Mesirov J.P. (2011). Integrative Genomics Viewer. Nat. Biotechnol..

[17] Yang H., Robinson P.N., Wang K. (2015). Phenolyzer: Phenotype-Based Prioritization of Candidate Genes for Human Diseases. Nat. Methods.

[18] Amberger J.S., Bocchini C.A., Schiettecatte F., Scott A.F., Hamosh A. (2015). OMIM.Org: Online Mendelian Inheritance in Man (OMIM^®^), an Online Catalog of Human Genes and Genetic Disorders. Nucleic Acids Res..

[19] Adzhubei I.A., Schmidt S., Peshkin L., Ramensky V.E., Gerasimova A., Bork P., Kondrashov A.S., Sunyaev S.R. (2010). A Method and Server for Predicting Damaging Missense Mutations. Nat. Methods.

[20] Pejaver V., Urresti J., Lugo-Martinez J., Pagel K.A., Lin G.N., Nam H.-J., Mort M., Cooper D.N., Sebat J., Iakoucheva L.M. (2020). Inferring the Molecular and Phenotypic Impact of Amino Acid Variants with MutPred2. Nat. Commun..

[21] Untergasser A., Cutcutache I., Koressaar T., Ye J., Faircloth B.C., Remm M., Rozen S.G. (2012). Primer3—New Capabilities and Interfaces. Nucleic Acids Res..

[22] Berglund J., Nevalainen E.M., Molin A.M., Perloski M., André C., Zody M.C., Sharpe T., Hitte C., Lindblad-Toh K., LUPA Consortium (2012). Novel origins of copy number variation in the dog genome. Genome Biol..

[23] Smit A.F.A., Hubley R., Green P. RepeatMasker Open-4.0. 2013–2015. https://www.repeatmasker.org.

[24] Perez G., Barber G.P., Benet-Pages A., Casper J., Clawson H., Diekhans M., Fischer C., Gonzalez J.N., Hinrichs A.S., Lee C.M. (2024). The UCSC Genome Browser database: 2025 update. Nucleic Acids Res..

[25] Blum M., Chang H.-Y., Chuguransky S., Grego T., Kandasaamy S., Mitchell A., Nuka G., Paysan-Lafosse T., Qureshi M., Raj S. (2021). The InterPro Protein Families and Domains Database: 20 Years On. Nucleic Acids Res..

[26] Bateman A., Martin M.-J., Orchard S., Magrane M., Ahmad S., Alpi E., Bowler-Barnett E.H., Britto R., Bye-A-Jee H., The UniProt Consortium (2023). UniProt: The Universal Protein Knowledgebase in 2023. Nucleic Acids Res..

[27] Piek C.J., Brinkhof B., Rothuizen J., Dekker A., Penning L.C. (2011). Leukocyte Count Affects Expression of Reference Genes in Canine Whole Blood Samples. BMC Res. Notes.

[28] Iio A., Motohashi T., Kunisada T., Yasuhira Y., Kamishina H., Maeda S. (2014). Preferential Gene Transcription of T Helper 2 Cytokines in Peripheral CCR4^+^CD4^+^ Lymphocytes in Dogs. Vet. Dermatol..

[29] Smith B.F., Stedman H., Rajpurohit Y., Henthorn P.S., Wolfe J.H., Patterson D.F., Giger U. (1996). Molecular Basis of Canine Muscle Type Phosphofructokinase Deficiency. J. Biol. Chem..

[30] Inal Gultekin G., Raj K., Lehman S., Hillström A., Giger U. (2012). Missense Mutation in PFKM Associated with Muscle-Type Phosphofructokinase Deficiency in the Wachtelhund Dog. Mol. Cell. Probes.

[31] Fu G., Liu H., Doerksen R.J. (2012). Molecular Modeling to Provide Insight into the Substrate Binding and Catalytic Mechanism of Human Biliverdin-IXα Reductase. J. Phys. Chem. B.

[32] O’Brien L., Hosick P.A., John K., Stec D.E., Hinds T.D. (2015). Biliverdin Reductase Isozymes in Metabolism. Trends Endocrinol. Metab..

[33] Karousis E.D., Mühlemann O. (2019). Nonsense-Mediated mRNA Decay Begins Where Translation Ends. Cold Spring Harb. Perspect. Biol..

[34] Boyko A.R., Quignon P., Li L., Schoenebeck J.J., Degenhardt J.D., Lohmueller K.E., Zhao K., Brisbin A., Parker H.G., vonHoldt B.M. (2010). A simple genetic architecture underlies morphological variation in dogs. PLoS Biol..

[35] Szydlowski M., Antkowiak M. (2022). No evidence that long runs of homozygosity tend to harbor risk variants for polygenic obesity in Labrador retriever dogs. J. Appl. Genet..

[36] Letko A., Hédan B., Snell A., Harris A.C., Jagannathan V., Andersson G., Holst B.S., Ostrander E.A., Quignon P., André C. (2023). Genomic Diversity and Runs of Homozygosity in Bernese Mountain Dogs. Genes.

[37] Letko A., Minor K.M., Jagannathan V., Seefried F.R., Mickelson J.R., Oliehoek P., Drögemüller C. (2020). Genomic diversity and population structure of the Leonberger dog breed. Genet. Sel. Evol..

[38] Papenhausen P.R., Kelly C.A., Harris S., Caldwell S., Schwartz S., Penton A. (2021). Clinical significance and mechanisms associated with segmental UPD. Mol. Cytogenet..

[39] Chen W., Maghzal G.J., Ayer A., Suarna C., Dunn L.L., Stocker R. (2018). Absence of the Biliverdin Reductase-a Gene Is Associated with Increased Endogenous Oxidative Stress. Free Radic. Biol. Med..

[40] Shaffer E.A. (2006). Epidemiology of Gallbladder Stone Disease. Best Pract. Res. Clin. Gastroenterol..

