# Supplementary material for: Biliverdinuria Caused by Exonic BLVRA Deletions in Two Dogs with Green Urine
Source: Genes (Basel). 2024 Nov 30;15(12):1561. doi: 10.3390/genes15121561 (PMC11675387; doi:10.3390/genes15121561)

Figure S1. Urine samples from Case 1 **(a)** and Case 2 **(b)** used for analysis of biliverdin and bilirubin concentrations.

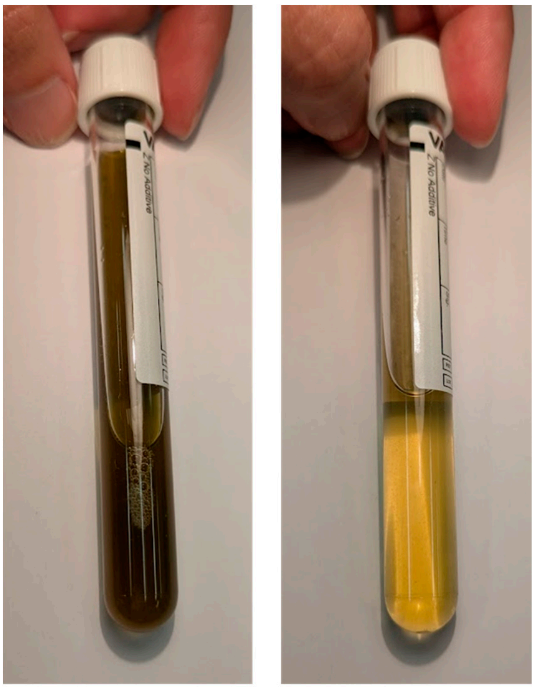

(a) **(b)**

Figure S2. Plots of intensity versus retention time (minutes) for biliverdin ("verdin") and bilirubin ("rubin") measured by liquid chromatography-tandem mass spectrometry in urine from Case 1 (**a**), Case 2 (**b**), and control dogs (**c-f**). Note that the y-axis limit for Case 1 is 30-fold greater than all other plots.

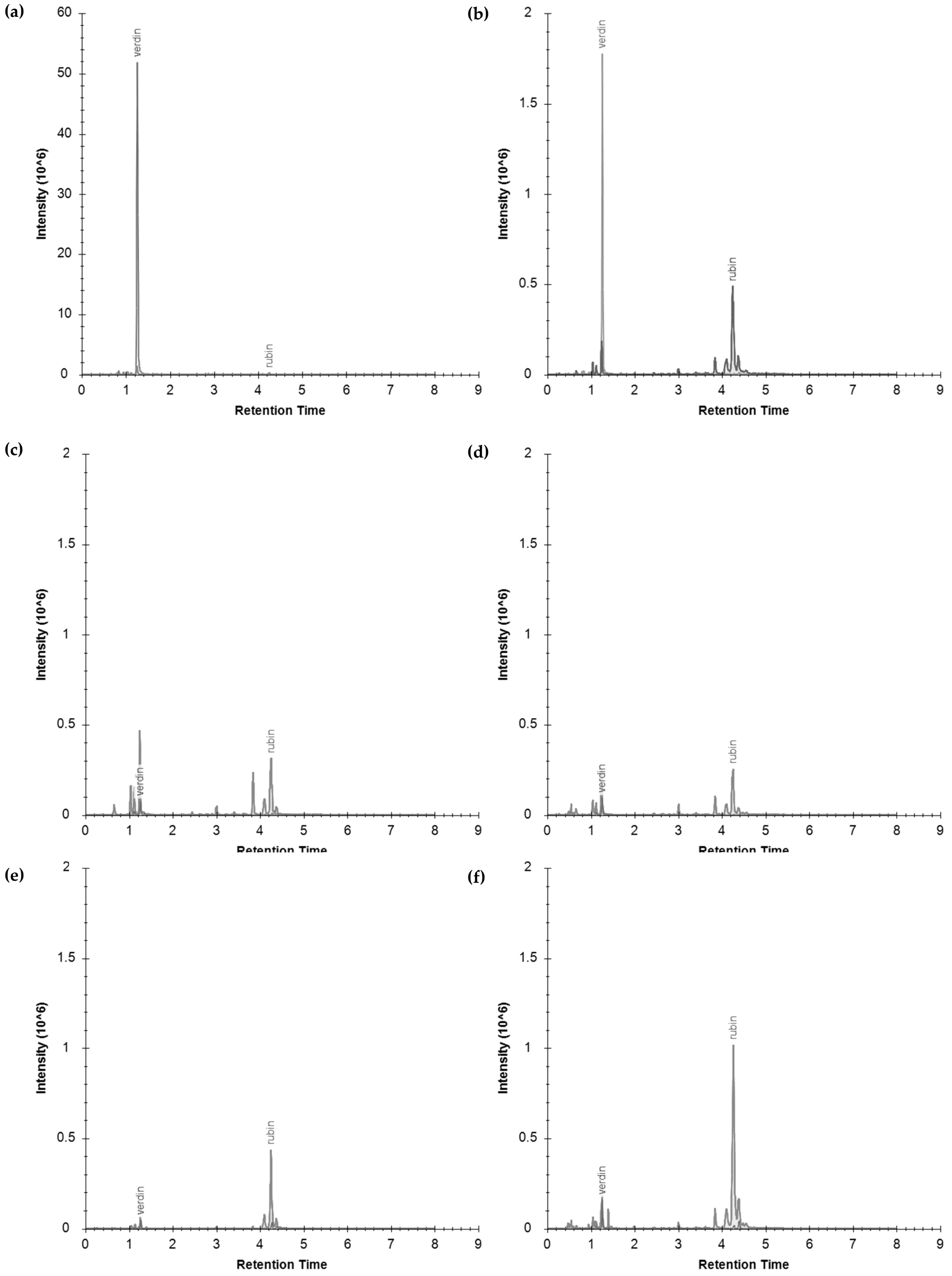

Supplement: Supplementary file 1 [file genes-15-01561-s001.zip › Biliverdinuria_Figures_S1-S2.pdf]
